# Knockdown of spliceosome U2AF1 significantly inhibits the development of human erythroid cells

**DOI:** 10.1111/jcmm.14370

**Published:** 2019-05-29

**Authors:** Jieying Zhang, Huizhi Zhao, Kunlu Wu, Yuanliang Peng, Xu Han, Huan Zhang, Long Liang, Huiyong Chen, Jingping Hu, Xiaoli Qu, Shijie Zhang, Lixiang Chen, Jing Liu

**Affiliations:** ^1^ Molecular Biology Research Center & Center for Medical Genetics, School of Life Sciences Central South University Changsha China; ^2^ School of Life Science Zhengzhou University Zhengzhou China

**Keywords:** erythropoiesis, spliceosome, U2AF1

## Abstract

U2AF1 (U2AF35) is the small subunit of the U2 auxiliary factor (U2AF) that constitutes the U2 snRNP (small nuclear ribonucleoproteins) of the spliceosome. Here, we examined the function of U2AF1 in human erythropoiesis. First, we examined the expression of U2AF1 during in vitro human erythropoiesis and showed that U2AF1 was highly expressed in the erythroid progenitor burst‐forming‐unit erythroid (BFU‐E) cell stage. A colony assay revealed that U2AF1 knockdown cells failed to form BFU‐E and colony‐forming‐unit erythroid (CFU‐E) colonies. Our results further showed that knockdown of U2AF1 significantly inhibited cell growth and induced apoptosis in erythropoiesis. Additionally, knockdown of U2AF1 also delayed terminal erythroid differentiation. To explore the molecular basis of the impaired function of erythroid development, RNA‐seq was performed and the Kyoto Encyclopedia of Genes and Genomes (KEGG) analysis results showed that several biological pathways, including the p53 signalling pathway, MAPK signalling pathway and haematopoietic cell lineage, were involved, with the p53 signalling pathway showing the greatest involvement. Western blot analysis revealed an increase in the protein levels of downstream targets of p53 following U2AF1 knockdown. The data further showed that depletion of U2AF1 altered alternatively spliced apoptosis‐associated gene transcripts in CFU‐E cells. Our findings elucidate the role of U2AF1 in human erythropoiesis and reveal the underlying mechanisms.

## 1 INTRODUCTION

Erythropoiesis is a process that erythrocytes are generated from haematopoietic stem cells (HSCs), including early erythropoiesis and terminal erythroid differentiation. During early erythropoiesis, HSCs proliferate and differentiate into burst‐forming‐unit erythroid (BFU‐E) and colony‐forming‐unit erythroid (CFU‐E) cells. During terminal erythroid differentiation, proerythroblasts undergo several mitoses to produce basophilic, polychromatic and orthochromatic erythroblasts. Orthochromatic erythroblasts enucleate to become reticulocytes, which subsequently mature into red blood cells.^1^, ^2^, ^3^, ^4^ The surface markers CD34, IL‐3R, CD36 and GPA have been defined for BFU‐E and CFU‐E cells in human early erythropoiesis.^5^ During terminal erythroid differentiation, the expression levels of GPA and band 3 increase and that of a4 integrin decreases.^6^ Thus, we used these surface markers to monitor the erythropoiesis process in vitro.

A series of genes and cell factors are involved in the regulation of erythroid differentiation. In contrast to extensive studies on the role of transcription factors and cytokines,^7^, ^8^, ^9^ little is known about the contribution of splicing to the regulation of normal erythropoiesis. The spliceosome is essential for removing noncoding introns from precursor messenger RNAs (pre‐mRNAs), which consist of the small nuclear ribonucleoproteins (snRNPs) U1, U2, U5 and U4‐U6.^10^, ^11^ Three essential sequences, the 5' splice site (5' ss), 3' splice site (3' ss) and branch point, are required for splicing. The process of spliceosome assembly is fully accomplished when a series of RNA splicing events are achieved. Splice site recognition is promoted by enhancers or prevented by silencers to regulate splicing throughout spliceosome assembly. Additionally, some specific enhancers and silencers are essential for the regulation of alternative splicing in the cell differentiation stage.^12^, ^13^, ^14^ The cytotoxic granule‐associated RNA binding protein T‐cell intracellular antigen 1 (TIA1) and TIA1‐like 1 bind to U‐rich motifs downstream of 5' ss in the regulation of the alternative splicing of exons.^15^ The RNA recognition motifs 1 (RRM1) and the C‐terminal glutamine‐rich (Q) domain are required for facilitating U1 snRNP recruitment to 5' ss. The Q‐rich domain of TIA1 interacts with the N‐terminal region of U1‐C to recruit U1 snRNP to 5' ss, and this process is enhanced by RRM1.^16^ A paralogue of U2AF65, RBM39, is involved in the pre‐mRNA splicing pathways in humans and Drosophila.^17^, ^18^

U2AF1 (also named U2AF35) is the U2 small nuclear RNA cofactor, which includes zinc finger domain, UHM and RS domain structures. Three conserved mRNA isoforms, termed U2AF1a (NM_006758), U2AF1b (NM_001025203) and U2AF1c (NM_001025204), were generated by alternative splicing of the U2AF1 gene. U2AF is a heterodimer composed of the small subunit U2AF1 and large subunit U2AF2 (U2AF65), among which U2AF1 is involved in the recognition of the AG at 3' ss and U2AF2 contacts the polypyrimidine tract.^19^, ^20^ U2AF1 binds to the AG dinucleotide interacting with both U2AF2 and SR protein to establish the earliest splicing complex E. Then, the U2AF complex replaces SF1 bind to the BPS with one of its subcomponents, SF3B1, to generate the splicing A complex.^21^ Mutations in spliceosome components have been detected in myelodysplastic syndromes (MDS). Reports on analysis of MDS patients have revealed a high incidence of U2AF1‐S34F missense mutation.^21^, ^22^ The study shows that overexpression of this U2AF1‐S34F mutant impairs erythroid differentiation and skews granulomonocytic differentiation towards granulocytes in human haematopoietic progenitors.^23^ Depletion of U2AF1 affects the alternative splicing of the Dscam gene transcript in Drosophila.^18^ Additionally, knockdown of U2AF1 alters alternatively spliced isoforms of Cdc25B and Cdc25C transcripts and retard mitotic progression in cancer cell.^24^, ^25^ Recently study revealed some spliceosome components, such as SF3B1 and SRSF2 deficiency impairs human erythropoiesis.^26^, ^27^ But the functions of depletion U2AF1 in erythropoiesis remain unknown.

To determine whether U2AF1 plays a key role in erythropoiesis, we knock down U2AF1 in erythroid cells derived from human CD34^+^ haematopoietic stem cells. We showed that depletion of U2AF1 impaired cell growth, led to apoptosis and delayed erythroid differentiation. Bioinformatics and biochemical analyses demonstrated that knockdown of U2AF1 induced apoptosis involving the p53 pathway and altered the alternative splicing of apoptosis‐associated gene transcripts. Our findings reveal a critical function of the U2AF1 and suggest that U2AF1 regulates alternative splicing during human erythropoiesis.

## 2 MATERIALS AND METHODS

### 2.1 Reagents and antibodies

Antibodies used for flow cytometry were PE‐CD235a (GPA), APC‐CD235a (GPA), PE‐CD34, FITC‐CD36, Percp‐Cy5.5‐7AAD and propidium iodide (PI) (BD Pharmingen); PE‐Cy7‐IL‐3R (CD123) and APC‐α4 integrin (Miltenyi Biotec); PE‐Cy7‐Annexin V (eBioscience); and human band 3 generated in our laboratory and labeled with FITC. Antibodies used for Western blotting were rabbit anti‐U2AF1 from Abcam; rabbit anti‐p53, rabbit anti‐p21, rabbit anti‐BBC3, rabbit anti‐BAX and rabbit anti‐Bcl‐2 from Cell Signalling Technology; anti‐actin antibody from Sigma; HRP‐conjugated mouse anti‐goat IgG from Invitrogen, and HRP‐conjugated goat anti‐rabbit IgG from Thermo Fisher.

### 2.2 Cell culture and lentivirus infection

Human peripheral blood samples were obtained from Xiangya Hospital of Central South University. Isolated CD34^+^ cells and sorted cells were cultured as previously described.^6^ HEK293T cells were plated in DMEM (Gibco) with 10% FBS (Gibco) at 37°C in 5% CO_2_. The control shRNA and two U2AF1 shRNAs were purchased from Sigma. All vectors were co‐transfected with the packaging vectors pCMV and pucMDG into HEK 293T cells for lentivirus production. The shRNA‐mediated knockdown was performed as previously described.^28^

### 2.3 Quantitative Real‐Time RT‐PCR

Total RNA was isolated from cells using the RNeasy mini kit (QIAGEN). For this procedure, 0.5 µg of RNA was synthesized into cDNA using ProtoScript First Strand cDNA Synthesis (New England Biolabs) according to the manufacturer's instructions. qRT‐PCR was performed using the AB17000 Sequence Detector (Applied Biosystems by Life Technologies) with Power SYBR Green PCR Master Mix (Applied Biosystems by Life Technologies). The relative gene expression was calculated using human ACTB (Qiagen) as an endogenous control. The primers were obtained from Harvard primer bank.

### 2.4 Flow cytometry and cell analysis

For cell cycle analysis, 0.5 × 10^6^ cells in 100 μL buffer (PBS + 0.5% BSA) were stained with PI and assessed by flow cytometry according to standard protocols. For analysis of apoptosis, 0.25 × 10^6^ cells were collected on day 6 and day 7 of culture and resuspended in 50 μL buffer. The cells were then stained with Annexin V and 7AAD according to the manufacturer's protocol. To monitor erythroid progenitor differentiation, 0.25 × 10^6^ cells in 50 μL buffer were stained with 7AAD, GPA, IL3R, CD34 and CD36. To monitor erythroid terminal differentiation, 0.25 × 10^6^ cells in 50 μL buffer were stained with 7AAD, GPA, Band3 and α4 integrin. For enucleation, 1 × 10^6^ cells were collected on day 13 and day 19 of culture and resuspended in 200 μL buffer (PBS + MgCl_2_). The cells were then stained with Syto‐16 and 7AAD according to the manufacturer's protocol (BD Pharmingen). Stained cells were analysed with a FACS Canto II (BD Pharmingen), and all data analysis was performed by FlowJo and BD FACSDiva software. For cell sorting, erythroid progenitor CFU‐E cells were stained as described above and sorted on a MoFlo high speed cell sorter (Beckman‐Coulter).

### 2.5 Colony assay

To determine the CFU‐E and BFU‐E potential, 200 cultured or sorted cells were resuspended in 1 mL of MethoCult classic medium (H4434) or MethoCult medium with EPO only (H4330) and plated in triplicate as previously described.^5^, ^29^ CFU‐E colonies were counted on day 7, and BFU‐E colonies were counted on day 14.

### 2.6 Western blotting

The cultured cells were harvested and washed with PBS, followed by the addition of RIPA buffer (Thermo Fisher) and protease inhibitor cocktail (Roche) for 30 minutes on ice and collection of protein extracts by centrifugation. The protein concentration was measured using a Pierce^®^ BCA protein assay kit (Thermo Fisher). Next, 40 µg of total protein was analysed by electrophoresis by 10% SDS‐polyacrylamide gel electrophoresis and transferred to a nitrocellulose membrane. The blots were blocked in 5% non‐fat milk and incubated with antibodies overnight at 4°C. The secondary antibody was purchased from Amersham Biosciences.

### 2.7 RNA‐seq and bioinformatics analysis

RNA‐sequencing was performed as previously described. RNA was extracted from sorted Luciferase control and U2AF1 knockdown CFU‐E cells. cDNA libraries were prepared using the Illumina TruSeq kit and sequenced using the Illumina HiSeq 4000 system. Quality control was performed before sequencing, and high‐quality reads from sequencing were aligned with Tophat2 following the detailed protocol.^30^ The cuffdiff and cummerbund R packages were used for the analysis and extraction of differentially expressed genes.^31^ Gene Ontology analysis and the Kyoto Encyclopedia of Genes and Genomes (KEGG) pathway were applied to annotate the biological functions.^32^ Cufflinks was used to assemble transcripts with the human hg19 genomes, and spliceR was used to annotate and extract differentially spliced genes.

### 2.8 Cytospin preparation

1 × 10^5^ cells in 100 μL 1 × PBS were spun on slides for 5 minutes at 300 rpm using the cytospin apparatus (Thermo Scientific). Then slides were stained with Giemsa solution (Sigma) according to manufacturer's instructions. The slides were analysed, and the images were acquired using a Leica DM2000 inverted microscope.

### 2.9 Statistical analysis

All data were processed and statistically analysed using SPSS 18.0 software. Significant differences between groups were determined using the *t* test, and *P* < 0.05 was considered to indicate a significant difference.

## 3 RESULTS

### 3.1 U2AF1 expressed during human erythroid differentiation

**Figure 1:**
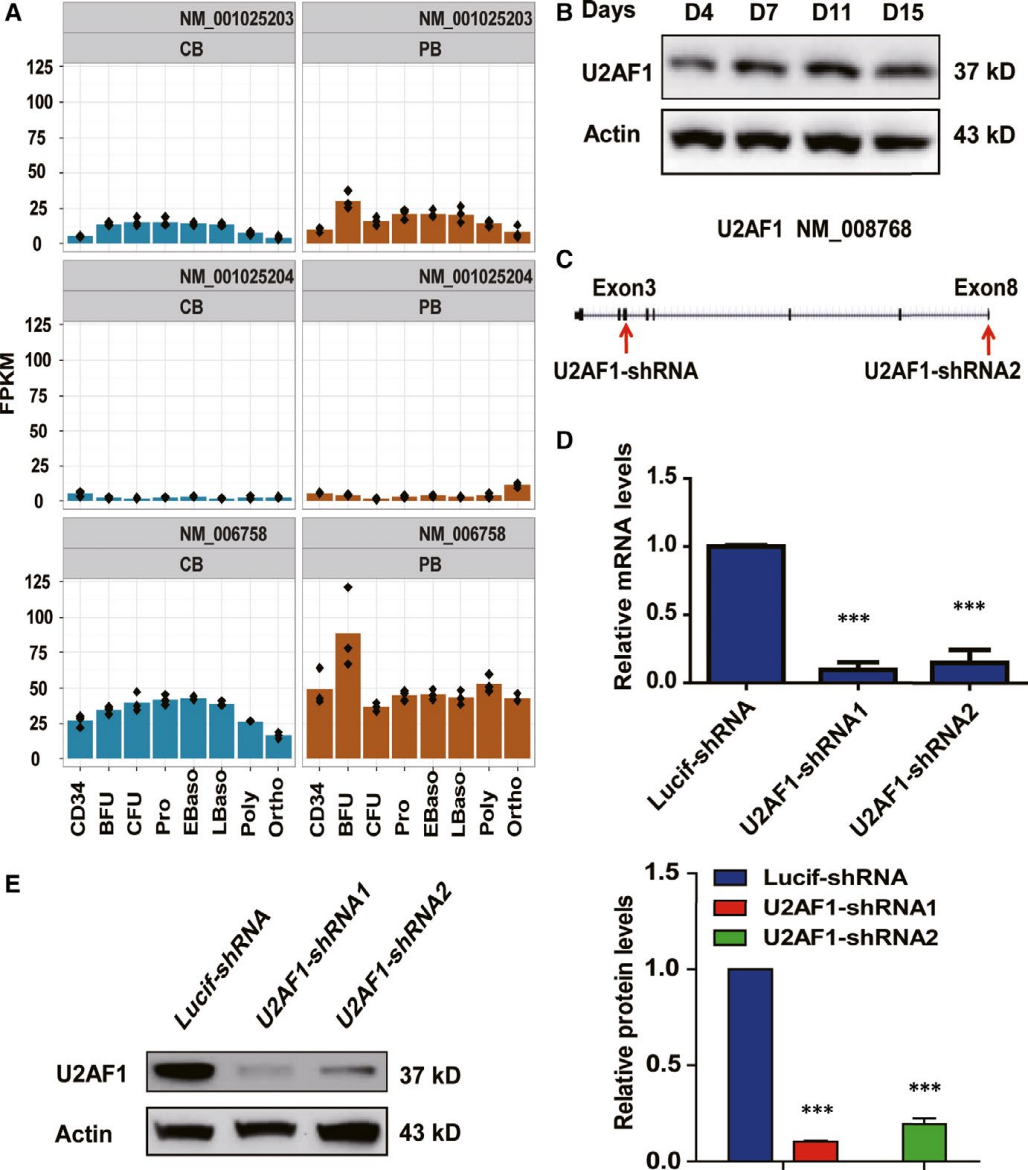
Expression of U2AF1 during human erythroid differentiation. A, RNA‐seq data showing the expression of three different isoforms of U2AF1 at each distinct stage of human erythroid differentiation. B, Representative image of Western blotting showing U2AF1 protein expression levels on days 4, 7, 11 and 15 of culture. C, Two U2AF1 shRNA target regions. D, qRT‐PCR results showing U2AF1 mRNA expression in erythroblasts transduced with lentivirus containing Lucif‐shRNA or U2AF1‐shRNA on day 6 of culture. Actin was used as an internal control. E, Representative image of Western blotting showing U2AF1 protein expression levels in erythroblasts transduced with lentivirus containing Lucif‐shRNA or U2AF1‐shRNA on day 6 of culture (left panel). Quantitative analysis of protein expression levels from three independent experiments is shown (right panel). Actin was used as a loading control. Statistical analysis of data from three independent experiments and bar plot representing means ± SD of triplicate samples. ***P < 0.001

Here, we analysed the expression of three different U2AF1 isoforms on the sorted cells during human erythropoiesis. The isoforms U2AF1a and U2AF1b were more abundant than U2AF1c, and the expression of U2AF1c was very low (Figure 1A). The expression of U2AF1a was higher in erythroid progenitor cells than other stages, and its expression was stable during terminal erythroid differentiation. The expression of U2AF1 was further confirmed at the protein level by Western blotting (Figure 1B). The expression of U2AF1 was knocked down using two different shRNA constructs (1 or 2) to target isoform a. The shRNA1 targeted the U2AF1a isoform‐specific exon 8 and shRNA2 target exon 3 (Figure 1C). The knockdown efficiency of U2AF1 shRNAs was confirmed by qRT‐PCR (Figure 1D) and Western blotting (Figure 1E).

### 3.2 Knockdown of U2AF1 impaired cell growth and increased apoptosis in early erythropoiesis

**Figure 2:**
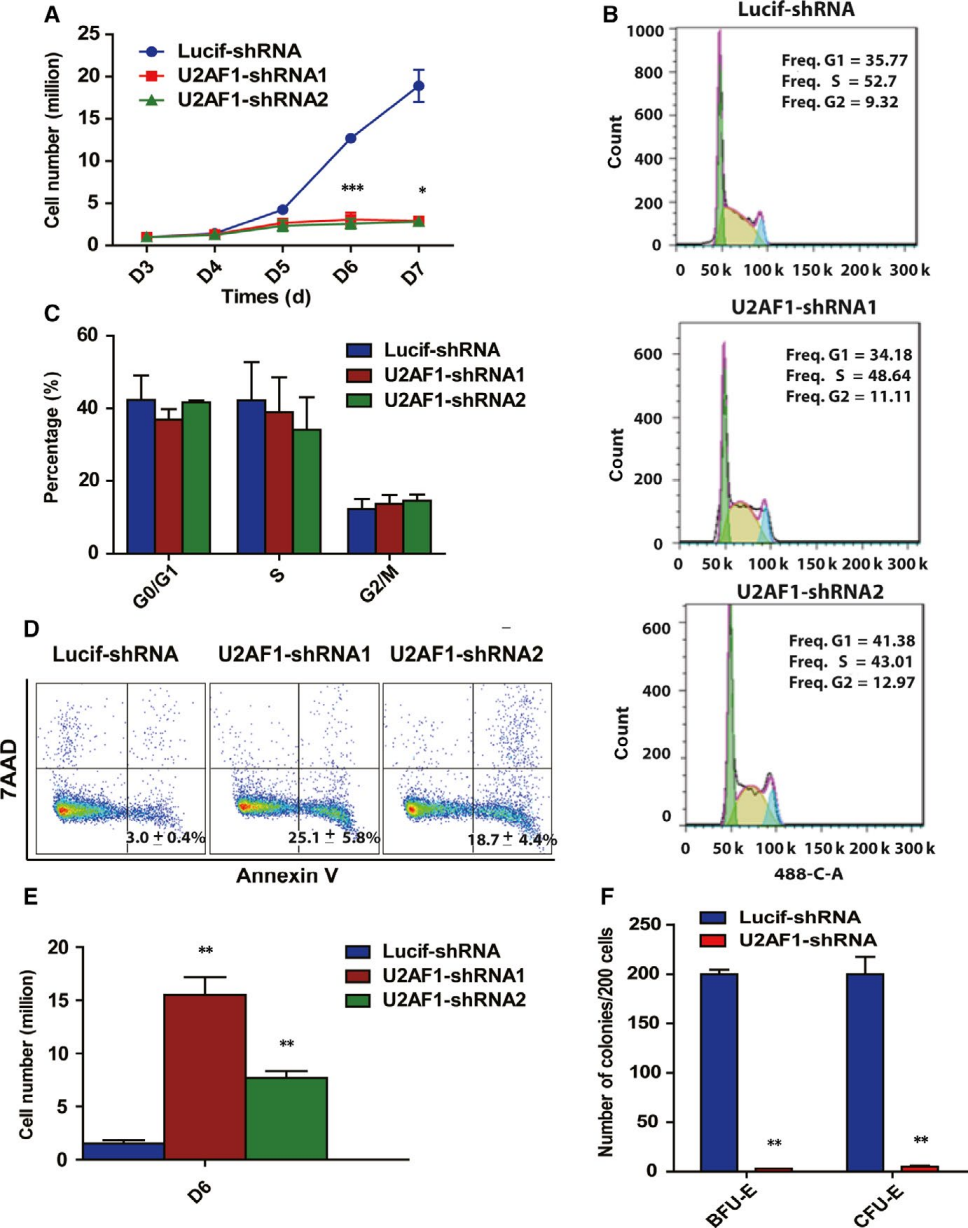
Effects of U2AF1 knockdown on the proliferation of human erythroid progenitors. A, Growth curves of cells transduced with lentivirus containing Lucif‐shRNA or U2AF1‐shRNA. B, Representative images of flow cytometry analysis of the cell cycle as assessed by propidium iodide and 7AAD staining of cells on day 6 of culture. C, Quantitative analysis of the cell cycle from three independent experiments is shown. D, Representative images of flow cytometry analysis of apoptosis by Annexin V and 7AAD staining on day 6 of culture. E, Quantitative analysis of apoptosis from three independent experiments on day 6 of culture. F, Colony forming ability of sorted burst‐forming‐unit erythroid and colony‐forming‐unit erythroid cells. Statistical analysis of data from three independent experiments and bar plot representing means ± SD of triplicate samples. *P < 0.05, **P < 0.01, ***P < 0.001

To understand the function of U2AF1 in early erythropoiesis, we transfected CD34^+^ cells with lentiviruses on day 2 and analysed them after day 3 of culture under our erythroid differentiation conditions. Cell growth was inhibited in U2AF1 knockdown cells at early stages compared with the control (Figure 2A). However, the PI staining results showed that U2AF1 knockdown did not affect cell cycle progression (Figure 2B,2C). The 7AAD and Annexin V staining and quantitative analysis revealed that the depletion of U2AF1 induced apoptosis (Figure 2D,2E). To examine whether U2AF1 was involved in the colony‐forming ability, we performed colony assays using sorted BFU‐E and CFU‐E cells. We then plated 200 cells on day 6 of culture in two different MethoCult media. Figure 2F shows that the BEU‐E and CFU‐E colonies decreased obviously in U2AF1 knockdown sorted cells compared with the control. Taken together, these data showed that U2AF1 knockdown impaired the proliferation of erythroid progenitors, induced apoptosis and reduced the colony forming ability.

### 3.3 Knockdown of U2AF1 impaired human terminal erythroid development

**Figure 3:**
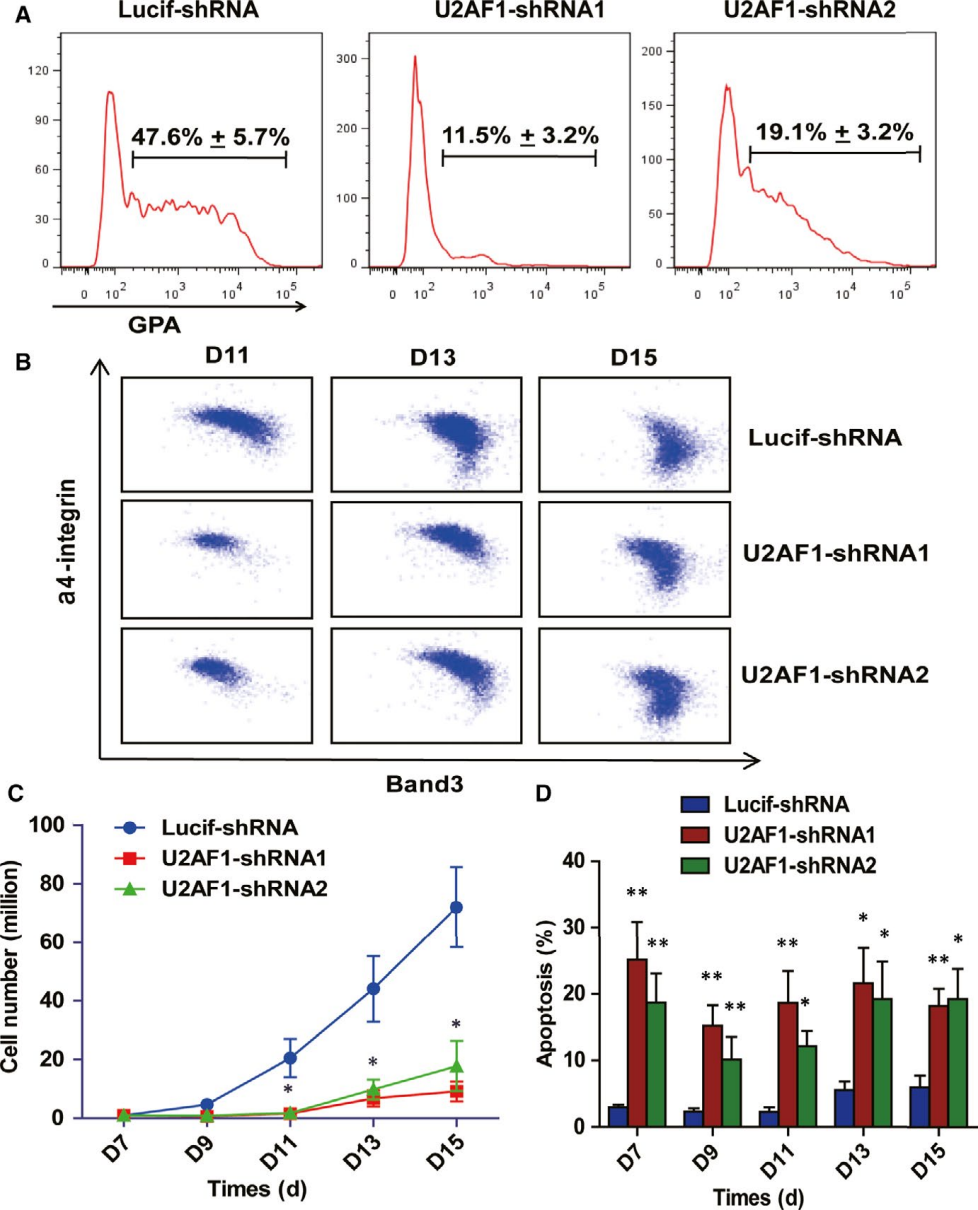
Effects of U2AF1 knockdown on terminal erythroid differentiation. A, Representative images of flow cytometry showing GPA expression in erythroblasts infected with Lucif‐shRNA or U2AF1‐shRNA on day 7 of culture. B, Flow cytometry analysis of band 3 and a4 integrin expression on different days in GPA+ erythroid cells infected with Lucif‐shRNA or U2AF1‐shRNA. C, Growth curves of erythroid cells transduced with LucifshRNA or U2AF1‐shRNA determined by cell counting. D, Quantitative analysis of apoptosis by Annexin V and 7AAD staining in erythroblasts infected with Lucif‐shRNA or U2AF1‐shRNA. Bar plot represents means ± SD of triplicate samples. *P < 0.05, **P < 0.01

Human terminal erythroid differentiation was monitored by flow cytometry based on the expression levels of surface markers.^33^, ^34^ As shown in Figure 3A, more than 45% of the Lucif‐shRNA‐transduced cells were GPA‐positive, while fewer than 20% of the U2AF1‐shRNA transduced cells were GPA‐positive. Furthermore, U2AF1 knockdown delayed terminal erythroid differentiation (Figure 3B). Next, we examined cell growth from day 7 to day 15 during terminal erythroid differentiation. The data showed that U2AF1 knockdown significantly impaired cell growth starting at day 7 of culture (Figure 3C). Additionally, the number of apoptotic cells increased in U2AF1‐shRNA‐transduced compared with Lucif‐shRNA‐transduced cells from day 7 to day 15 (Figure 3D).

### 3.4 Knockdown of U2AF1 impairs enucleation and leads to the generation of erythroblasts with abnormal nucleus

**Figure 4:**
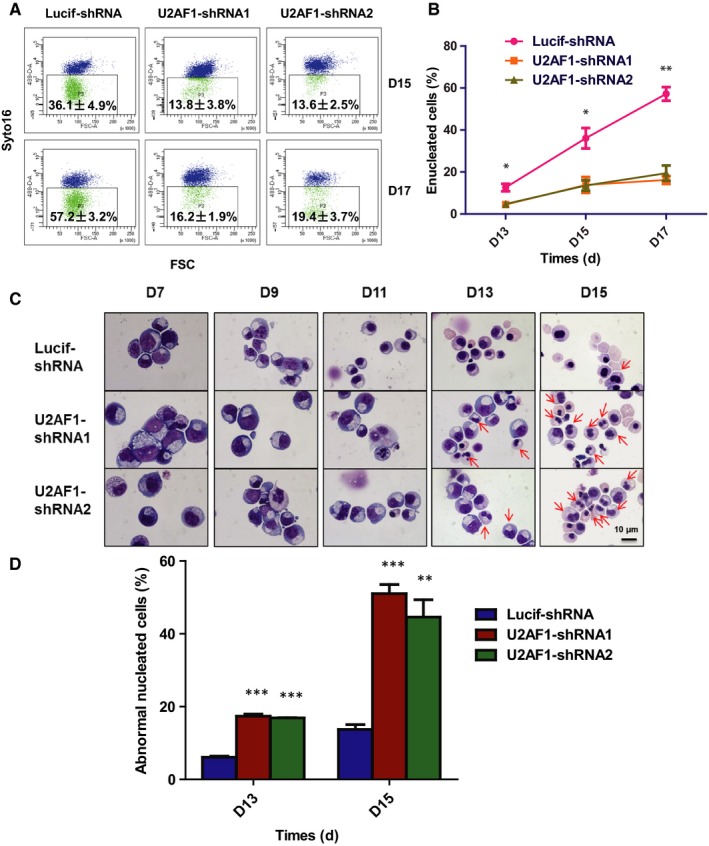
Knockdown of U2AF1 impairs enucleation and leads to the generation of erythroblasts with the abnormal nucleus. A, Representative profiles of enucleation as assessed by Syto‐16 staining on day 15 and day 17 of culture. B, Quantitative analysis of enucleation on indicated days from three independent experiments. C, Representative cytospin images of erythroblasts cultured for 15 d. D Quantitative analysis of abnormally nucleated erythroblasts at day 13, day 15 of culture from three independent experiments. *P < 0.05, **P < 0.01, ***P < 0.001

The effect of U2AF1 knockdown on enucleation, we assessed the enucleated cells from day 13 to day 19 and Figure 4A shows the representative profiles. Quantitative analysis reveals that the Lucif‐shRNA‐transduced cells started to enucleate on day 13 and more than 60% of cells enucleated by day 19. In contrast, the enucleation rate of U2AF1‐shRNA2 transduced cells was fewer than 20% on day 19 (Figure 4B). When examining the effect of U2AF1 knockdown on cell morphology, we found that many erythroblasts exhibited abnormal nuclei at the late stage. Cytospin analysis of cultured erythroblasts revealed the generation of abnormal nuclei occurred specifically at day 13 and day 15 of culture (Figure 4C). Quantitative analysis showed that approximately 20% of erythroblasts at day 13 of culture and 40%‐50% of erythroblasts had abnormal nuclei at day 15 of culture compared to control (Figure 4D).

### 3.5 Knockdown of U2AF1 affected gene expression in erythroblasts

**Figure 5:**
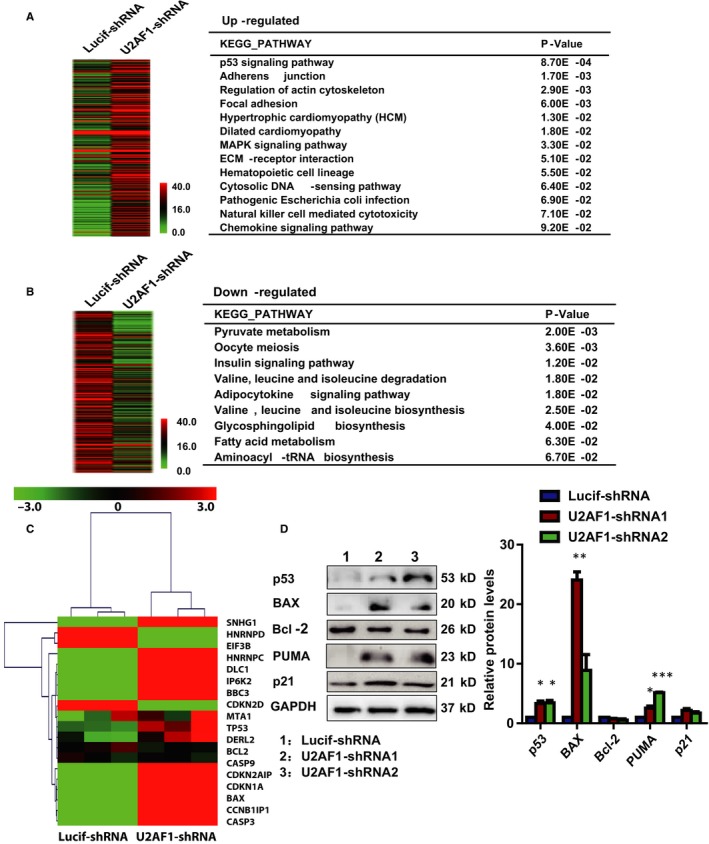
Knockdown of U2AF1 affected gene expression in colony‐forming‐unit erythroid (CFU‐E) cells. RNA‐seq data were processed by the BGI standard and showed differentially expressed genes between Lucif‐shRNA and U2AF1‐shRNA. The differentially expressed genes were analysed as an FDR < 0.05 and fold change ≥2. Hierarchical clustering of differentially expressed genes that were up‐regulated at least 2.0‐fold (A) and down‐regulated at least 2.0‐fold (B) between Luciferase and U2AF1 knockdown CFU‐E cells in three independent experiments. Every gene is coloured according to its average mRNA expression level. The differentially expressed genes involved in the pathway are indicated in the right panel. C, Heatmap showing the GSEA analysis of the differentially expressed genes between Luciferase and U2AF1 knockdown CFU‐E cells. D, Representative images of Western blotting showing the expression of p53, BAX, Bcl‐2, BBC3 and p21 in erythroblasts on day 6 of culture (left panel). Quantitative analysis of protein expression levels from three independent experiments is shown (right panel). *P < 0.05, **P < 0.01, ***P < 0.001

To further explore the underlying molecular mechanisms for the changes in cell growth and apoptosis, we sorted Luciferase‐knockdown and U2AF1‐knockdown CFU‐E cells for RNA‐seq analysis. There were 628 differentially expressed genes, including 282 up‐regulated (Figure 5A) and 346 down‐regulated genes (Figure 5B), between Luciferase‐knockdown and U2AF1‐knockdown CFU‐E cells. Using the KEGG pathways to analyze the 628 differentially expressed genes, we found that these genes were involved in several biological pathways, including the p53 signalling pathway, MAPK signalling pathway and haematopoietic cell lineage. The data from microarray analysis showed that many of the differentially expressed genes were involved in the p53 signalling pathway (Figure 5C). The p53‐induced apoptosis is mediated by transcriptional activation of Bcl‐2 family genes such as Bcl‐2, BAX and BBC3.^35^, ^36^ Western blot analysis revealed increased protein levels of p53, BAX, PUMA (BBC3) and p21 and decreased protein levels of Bcl‐2 in U2AF1‐knockdown CFU‐E cells (Figure 5D).

### 3.6 Knockdown of U2AF1 affected the alternative splicing of genes in erythroblasts

**Figure 6:**
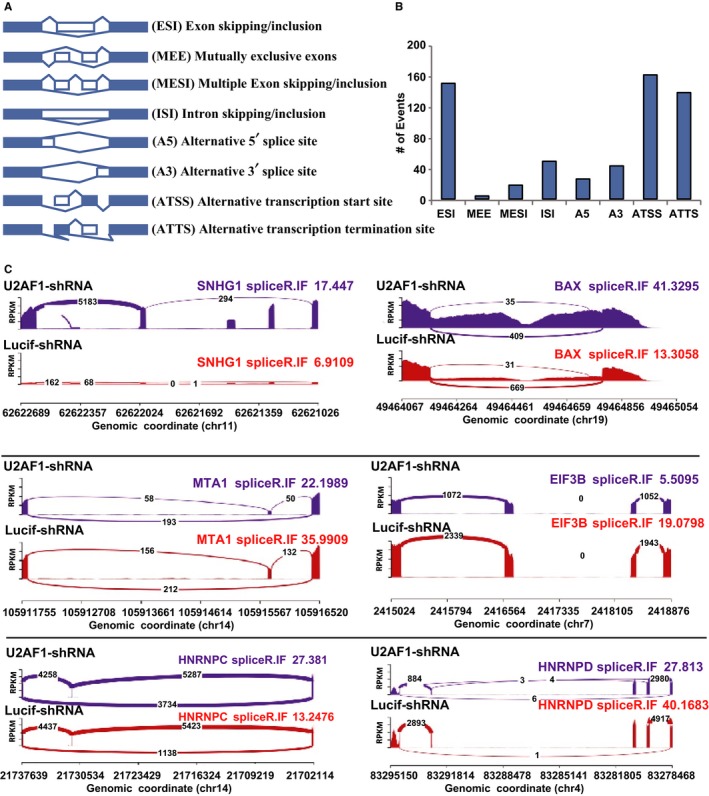
Knockdown of U2AF1 affected the alternative splicing of apoptosis‐associated genes in CFU‐E cells. A, The kinds of splicing events. B, The most frequent splicing events after U2AF1 knockdown: exon skipping/inclusion (ESI), multiple exon skipping/inclusion (MESI), intron skipping/inclusion (ISI), alternative 5’ splice site (A5), alternative 3’ splice site (A3), alternative transcription start site (ATSS), alternative transcription termination site (ATTS) and mutually exclusive exons (MEE). C, Schematic expression of differentially spliced transcripts.

Previous research has indicated that U2AF1 is a regulator of alternative splicing and is involved in recognition of the AG at 3' ss. To study the effect of U2AF1 on alternative splicing, we analysed the RNA‐seq data and found that 543 transcripts were differentially spliced in Lucif‐shRNA and U2AF1‐shRNA CFU‐E cells. There were eight kinds of splicing events (Figure 6A), and notable splicing events were exon skipping/inclusion (ESI), alternative transcription start site (ATSS) and alternative transcription termination site (ATTS) in CFU‐E cells (Figure 6B). To monitor alternative splicing, we analyzed the SNHG1, BAX, MTA1, EIF3B, HNRNPC and HNRNPD genes, which play an important role in apoptosis. As shown in Figure 6C, depletion of U2AF1 affected alternative splicing of these genes transcripts compared with Lucif‐shRNA‐transduced cells.

## 4 DISCUSSION

RNA splicing is an important post‐transcriptional process during which introns are removed and exons are retained from pre‐mRNA by spliceosomes. The U2AF family members U2AF1 and U2AF2 have been implicated in the regulation of splicing. It has been previously reported that the expression of the U2AF1a isoform is more abundant than U2AF1b in higher vertebrates. Additionally, the U2AF1c isoform is targeted by RNA surveillance for the introduction of a premature termination codon.^37^ In addition, studies have demonstrated that U2AF1a is essential for HeLa cell division and that knockdown of U2AF1a, alone or in conjunction with U2AF1b, has a more significant effect than knockdown of U2AF1b alone.^24^ Most studies have been demonstrated mutational analysis of U2AF1 splicing machinery in haematological malignancies and other tumours. However, very few studies have focused on the cellular functions of U2AF deficiency.

Mutations of U2AF1 have been implicated in MDS patients with MDS disease without an increase in ring sideroblasts (MDS without RS), chronic myelomonocytic leukaemia, de novo AML and myeloproliferative neoplasms.^21^ U2AF1*‐*S34F impaired erythroid differentiation and reduced growth of erythroid progenitors in human haematopoietic progenitors. Expression of U2AF1‐S34F in human haematopoietic progenitors leads to the skewing of granulo‐monocytic differentiation towards granulocytes.^23^ Different phenotypes were observed in ex vivo cultures of primary human CD34^+^ cells model, U2AF1 mutations (S34F, Q157R) skew granulo‐monocytic differentiation towards monocytes. During human HSPCs megakaryo‐erythroid differentiation, expression of U2AF1‐S34F and U2AF1‐Q157R resulted in a decrease in the megakaryocyte with a compensatory increase in the erythroid precursors populations.^27^ However, it has remained unclear whether depletion of U2AF1 plays any vital role in human erythropoiesis. We analysed the expression of three isoforms of U2AF1 on the sorted cells during human erythropoiesis. Our results showed higher expression of U2AF1 in early erythropoiesis than in terminal erythroid differentiation. In addition to increased apoptosis, U2AF1 knockdown also impairs enucleation and results in generation of late‐stage erythroblasts with abnormal nuclei. This may be due to the expression of cytokinesis‐/ mitosis‐associated genes are affected by U2AF1 depeletion. Knockdown of U2AF1 impaired the growth of primary erythroid progenitor cells. These findings demonstrated a critical role for U2AF1 in haematopoiesis progenitor cells. Furthermore, our results showed that the cell cycle was not affected and that apoptosis increased following the U2AF1 knockdown, suggesting that the failure to grow was due to apoptosis. We found that depletion of U2AF1 impaired the proliferation of HSCs and induced apoptosis during granulo‐monocytic differentiation. Morphological observation showed that inhibition of U2AF1 expression caused a significant decrease in generation of macrophage (data not show). These variations could be caused by the differences in sample source and cell culture conditions, including the presence of serum, and combinations of cytokines. During erythroid differentiation, knockdown of U2AF1 inhibited cell growth and delayed differentiation. The molecular mechanisms responsible for the inhibited growth and induced apoptosis in U2AF1 knockdown‐erythroid cells appeared to include U2AF1 knockdown‐affected gene expression. The differentially expressed genes were analysed by functional annotation, and the results showed that these genes were involved in several biological pathways including the p53 signalling pathway, MAPK signalling pathway, haematopoietic cell lineage and metabolic pathway. Western blotting analysis revealed an increase in the expression levels of p53 and its downstream targets. Activation of the p53 pathway has previously been shown to impair erythropoiesis in mouse and human model systems.^38^, ^39^, ^40^

U2AF1 is a core component of the mRNA splicing machinery, and its depletion may cause abnormal pre‐mRNA processing of transcripts encoding proteins that are critical for the regulation of cell growth and apoptosis. U2AF1‐S34F induced a higher number of cassette exon splicing events in granulo‐monocytic cells than in erythroid cells.^23^ In A549 lung cancer cells, both U2AF1 knockdown and mutantion lead to distinct alternative splicing patterns. Ectopic expression of U2AF1mutation but not knockdown was shown to result in common alternative splicing events involved in cell cycle progression.^25^ Consistent with this hypothesis, we documented that a deficiency of U2AF1 altered splicing pattern. In contrast to the Luciferase control, U2AF1 knockdown cells had decreased levels of spliced transcripts encoding MTA1, EIF3B, HNRNPC and HNRNPD and increased levels of BAX and SNHG1. MTA1 regulated p53 stability and decreased p53‐mediated apoptosis, which is also involved in p53 signalling pathways in prostate cancer (PCa) cells.^41^, ^42^ Furthermore, depletion of MTA1 increases levels of Res‐induced apoptosis and the accumulation of Bax in PCa cells.^43^ EIF3B knockdown decreases cell viability and increases apoptosis.^44^, ^45^ HNRNPD is a p53 target, and SNHG1 increases p53‐dependent apoptosis by impairing hnRNPC regulation of p53 activity.^46^, ^47^ In this study, the splicing patterns of these gene were found to be ESI, ISI, ATSS and ATTS. Our findings have implications for the understanding of the relationship between splicing factors and disordered erythropoiesis such as MDS.

## CONFLICT OF INTERESTS

The authors declare no conflict of interest.

## AUTHOR CONTRIBUTIONS

JZ, YP, XH, HZ, LL, HC, JH, XQ, SZ and LC performed the research and analysed the data. HZ and KW performed the bioinformatics analysis. JL designed the experiments, analysed the data and wrote the paper. All authors read and approved the final manuscript.
